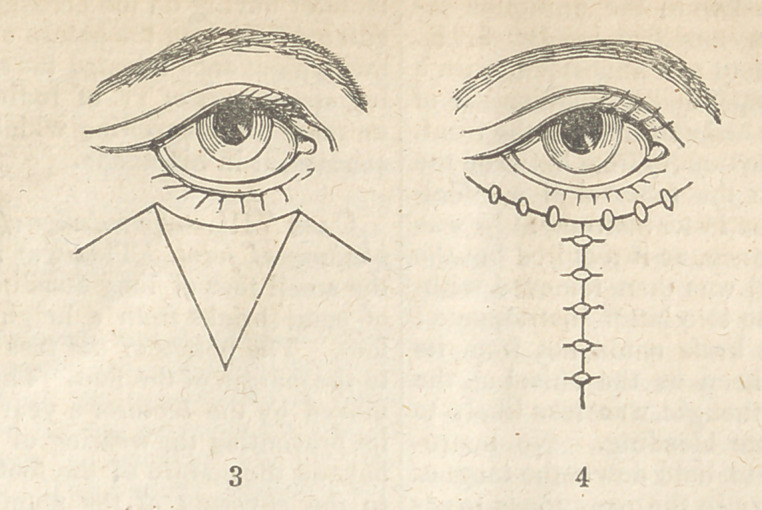# Jefferson Medical College

**Published:** 1843-02-04

**Authors:** H. T. Child


### JEFFERSON MEDICAL COLLEGE.

### CLINIC OF PROFESSOR MÜTTER.

*Dispensary of Jefferson Medical College, Jan. 11, 1843.*

(Reported by H. T. Child.)

Case I.—*Ulcer of the lid, of seven years standing—cause unknown—Blepheroplastic operation for its relief—results of operation.*—Professor Mütter commenced the lecture of this morning by exhibiting the results of a very extensive blepheroplastic operation, performed before the class about five weeks since. This patient was a woman, aged about 40, who, for several years had suffered from a chronic ulcer, partaking somewhat of the nature of lupus, but involving the integument only, and which occupied nearly the whole of the lower lid of the left side. All the usual remedies having failed to cure the disease it was determined to perform the operation alluded to above.

The object being to remove the whole of the diseased tissue and substitute for it a flap of healthy skin, the operation was commenced by making an incision, starting from the inner canthus, and continued downwards and outwards, until it ceased about an inch and a half below the inferior orbitar ridge; another incision was then carried from the external canthus downwards and forwards, to meet the first; the whole of the diseased mass was thus included between the two, and then immediately dissected out, leaving a space of the shape of the letter V.

The oozing of the blood having in a great measure ceased, and the parts being carefully examined, in order to prevent the least particle of the diseased tissue being left behind, the second step of the operation was commenced. Starting from the superior extremity of the external incision, the bistoury was made to take a course, at first upwards for about six lines, and then outwards for an inch and a quarter, the latter being curvilinear, in order that the upper margin of the flap might correspond in shape to the natural curve of the eyelid. *(See fig. 1.)*

1

2

From the terminal extremity of the incision another was carried downwards and forwards, until it reached a point opposite the union of the two first. The flap included in these incisions was then dissected up, brought over the raw surface from which the diseased tissue had been removed, and attached to the sound skin by two twisted and two interrupted sutures. (*Fig.* 2.) The surface from which it had been removed was then closed, by drawing the edges of the wound together by the twisted suture and straps. A compress was then applied over the flap and secured with a roller, *firmly* applied, so as to prevent by pressure, the oozing of blood, which, in all operations of this kind, is one of the chief obstacles to union by the first intention. The patient was then ordered to be kept quiet, the compress to be saturated with cold water; the head to be maintained in an elevated position, and the diet to be absolute.

Nothing of interest occurred in the subsequent treatment, and in about two weeks the patient was perfectly cured, union by the first intention having taken place throughout. The surface from which the flap had been taken, united in the same way with the exception of a small space a few lines in diameter, which healed by granulation.

The appearance of the patient is greatly improved, and the operation may be considered as perfectly successful.

Professor Mütter remarked that many surgeons had no confidence in plastic surgery, from the fact that the flap generally shrinks, and in consequence of this the patient is often as much deformed as before he submitted to the operation. But from very ample experience he was able to state that this result was to be attributed, rather to the surgeon than to the operation, for if the flaps are made large, much larger than would seem necessary at the time, when contraction takes place, there is enough tissue to spare, and at the same time to cover in completely the parts upon which it has been applied.

No operation of this class is more deserving our confidence than Blepheroplasty, (from Βλέφαρον, the eyelid, and πλαστικη,) especially when the lower lid is the seat of the disease; in affections of the upper lid its results are less satisfactory.

The deformities and diseases of the lids requiring the performance of this operation, are ectropium, entropium, ulcerative degeneration, destruction of the organ, and its transformation into innodular tissue, so often met with as a result of severe burns or scalds of the face. In the older works which treat of plastic surgery, this particular operation is not mentioned, and even Celsus lays it down as an axiom that a lid once lost cannot be restored: "*Si pelpebra tota deest nulla id curatio restiture potest.*" It was first performed in 1816 by Græfe. The complex organization and delicacy of the tissue to be restored, render blepheroplasty an operation of great interest and difficulty, and it is impossible, as Zeis well observes, to produce an artificial lid from the common integuments which shall possess mucous membrane, muscle, glands, ciliæ, cellular tissue, &c. &c., but we can imitate nature very closely, in the *form* of the protection, which it is our object to supply to the tender organ that has been deprived of its natural covering.

It is really surprising how much, in this respect, can be done, and the case before us is a good illustration of this fact, for it is almost impossible to say on which eye the operation has been performed.

The surface of the flap next to the ball has been converted into a tissue similar, in most respects, to mucous membrane; no adhesions exist between it and the ball, and hence the movements of the latter are free—the tears pass into the punctum lacrymale as usual, and the margin of the flap is rounded off so as to resemble a natural lid.

Various plans have been adopted to carry into effect the object of the surgeon, but it must be obvious that no one method is appropriate to every case, nor will it be proper to attempt the execution of any plastic operation, unless there exists in the vicinity of the eye, an abundant supply of healthy integument and subcutaneous cellular tissue.

In cases of simple eversion of the lid from any cause not malignant, Dzondi proposed to "divide the cicatrix and allow it to heal by granulation, so that the broader scar might remedy the defect." A very strong objection to this operation at once presents itself in the fact that the incision, in healing will contract, and may in consequence, of this, increase the deformity. Nor is the operation of Sir William Adams, the one chiefly in vogue, applicable to any other cases than those in which there exists simple eversion of the lid, without much alteration in its structure, and wherever the entire organ is to be restored nothing short of plastic surgery will prove of the least utility.

The operation of Dieffenbach for ectropium of the lower lid is much more to be relied on. He removes the cicatrix by an incision of a triangular shape, the basis of which is towards the ciliary margin, and the apex downwards; he next divides the integuments laterally, the incision being curvilinear, then cuts out the diseased tissue, and finally raises the lateral portions forming the sides of the triangle; the cuts margins are then brought together, and united by suture. *(Fig. 3 and 4.)*

3

4

The operation of Fricke, of Hamburg, for making a lid has many advocates, among whom are Jünghen, Blanden, Gerdy, Jobert, Carron, Liston, Warren, and, I believe, Kearney, Rodgers, and Post, of New York; and is undoubtedly an operation to be relied upon in many cases, although my own experience with it leads me to prefer another plan. This operation consists in cutting away the diseased mass, or dividing the lid, so that its ciliary margin may be sparated from the lower portion, and thus leave a space between them. Into this space, or upon the raw surface, where the whole lid is removed, a flap of integument of the proper shape and size, taken from the temple, is then placed, and attached by sutures to the edges of the wound.

The objection to this method is, the necessity always existing for *torsion* of the *pedicle* of the flap, by which the probability of union is much diminished, and the danger of sloughing from a want of blood increased.

Another method of operating has been proposed by Dieffenbach, which deserves our highest confidence, and must undoubtedly become the favourite operation of every one where the case admits of its being carried into execution. It has already been repeated by Professor Ammon of Dresden, Zeis, Von Ekstrom, Fricke, Peters, Lawrence, Tyrrell, and others, and is the one performed in the case now presented, which makes the third in my own practice.

The advantages of this method, which belongs to plastic operations by "*inclination* of the *flap*," are, the facility with which it is executed, the little risk of sloughing from the pedicle of the flap being scarcely, if at all, subjected to torsion, and the trifling scar which it leaves.

Professor Jaeger, of Berlin, has proposed a plan of operating that may do very well in some cases, and therefore I will explain it to you.

The operation belongs to the class of plastic operations by *sliding the flap*, (glissement du lambeau,) and is peculiarly adapted to cases of lagophthalmos and ectropium. It consists in first cutting through the everted or shortened lid in its whole thickness by a transverse incision, including its whole breadth; he then cuts out a perpendicular piece, so as to bring the lid to its proper width; he next loosens the integuments of the cheek, if he is operating upon the lower lid, or of the forehead if the upper is the seat of disease, with a double edged knife carried between the orbicularis muscle and the bone, so that they can be drawn upwards or downwards to a sufficient extent. The wounds are then united by sutures. A method somewhat similar to this has been proposed by T. W. Jones, of England. He includes the cicatrix or contracted portion in two incisions, which unite at an acute angle, so as to form a V, and must extend into sound tissue. He then draws upon the flap, so as to stretch out the cellular tissue beneath it, and when this does not yield readily, he dissects up a portion of the flap; and, by thus sliding the skin, he expects to gain the object in view. This operation might possibly answer in some cases, but I do not recommend it as we have other means more worthy of confidence, and it could rarely, if ever, be performed where it is necessary to form the entire lid.

Professor Horner has recently performed an operation for ectropium, which differs a little from the operations mentioned, but belongs to the class of *operations by "displacement of the flap."*

Professor Pancoast has recently reported two cases of blepheroplastic operation, in which he combined the methods of Dieffenbach, Sir William Adams, and Jones, and in both cases his success was complete.

(To be continued.)

## Figures and Tables

**Figure f1:**
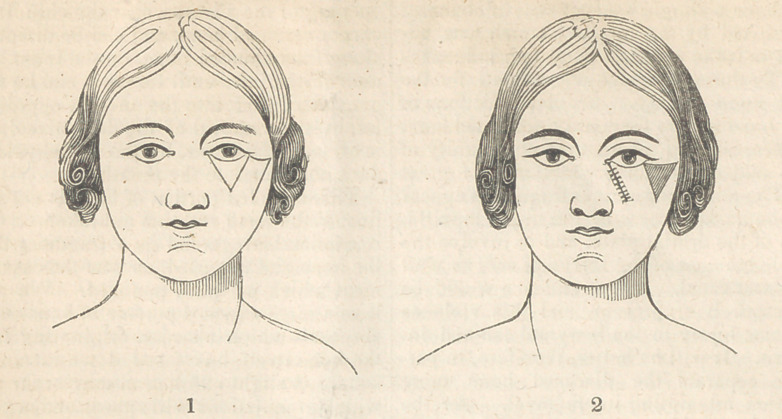


**Figure f2:**